# The Benchmark That Keeps Losing: What Federal Arbitration Is Revealing About the Qualifying Payment Amount

**DOI:** 10.7759/cureus.114331

**Published:** 2026-08-11

**Authors:** Andrew M Klapper, Anthony N Dardano, Michael Risin, Taylor Florio, Martha Denavea

**Affiliations:** 1 Plastic and Reconstructive Surgery, Delray Medical Center, Delray Beach, USA; 2 Nursing, Revenue Cycle, and Clinical Documentation, Floridian Institute of Plastic Surgery, Delray Beach, USA

**Keywords:** arbitration, emergency care, health policy, independent dispute resolution, no surprises act, out-of-network reimbursement, payer transparency, payment enforcement, qualifying payment amount, reimbursement policy

## Abstract

This editorial examines the role of the qualifying payment amount in federal independent dispute resolution under the No Surprises Act. It distinguishes patient protection from payment valuation, evaluates competing interpretations of federal dispute outcomes, and considers procedural and enforcement concerns associated with the dispute-resolution process. It proposes three reforms: confidential verification of qualifying payment amount inputs, searchable carrier-level outcome reporting, and enforceable consequences for untimely payment of awards. The editorial argues for independent examination of the benchmark while preserving patient protections and neutral review.

## Editorial

The No Surprises Act (NSA) achieved an essential goal: patients are generally protected from balance billing when they unknowingly receive covered out-of-network emergency services or certain out-of-network services at in-network facilities [1]. That protection was overdue and should remain intact.

Patient protection, however, is not the same question as payment valuation. Congress removed the patient from the dispute and substituted a federal independent dispute resolution (IDR) process between providers and payers. At the center of that process sits the qualifying payment amount (QPA), defined generally as an insurer's median 2019 in-network contracted rate for the same or similar item or service, indexed forward [1,2]. The QPA is not an independently observed market price. It is a statistic drawn from contracts negotiated for network participation and then applied to clinicians who did not sign them.

The physician furnishing noncontracted emergency care did not sign those contracts, yet those contracts anchor the payment discussion. That is the central contradiction. The QPA may not be wrong in every case, but it cannot reasonably be presumed right simply because the payer calculated it.

What the federal data establish and what they do not

The national results are difficult to ignore. In 2024, providers and facilities prevailed in approximately 85% of federal payment determinations. Importantly, providers also prevailed in approximately 85% of nondefault determinations, meaning the pattern persisted in contested disputes in which both sides submitted offers. Approximately 85% of selected offers exceeded the QPA, while insurer offers were at or below the QPA in roughly 47% of disputes [2]. For emergency-service disputes, median prevailing offers in 2023 and 2024 were approximately 2.5-3 times the QPA [3].

Those figures do not establish an objectively correct price. IDR cases are selected, not random. Providers and specialized representatives screen claims before filing; weaker or lower-value disputes may never be pursued. Baseball-style arbitration also chooses the more reasonable of two submitted offers. It does not calculate a true market-clearing price, and both offers could theoretically be high or low. A provider victory may reflect case acuity, documentation, offer strategy, insurer default, or an inadequately supported payer offer rather than a direct rejection of the QPA itself.

The limitations matter, but they do not erase the pattern. They define the inference that can responsibly be drawn: the outcomes do not validate every provider offer, but they are more than sufficient to reject automatic deference to the QPA. When neutral entities repeatedly select values above the benchmark across a large number of contested cases, the benchmark stops being an article of faith and becomes the obvious subject of investigation.

The most defensible conclusion is comparative, not absolute. Federal IDR outcomes show persistent divergence between the QPA and selected payment offers. They do not, by themselves, identify whether the divergence reflects QPAs that run low, provider offers that run high, or some combination. That is precisely why independent, carrier-level analysis is needed (Table 1).

**Table 1:** Summary of federal IDR outcome and cost data cited in this editorial Values are approximate and reflect federal IDR determinations reported by the Congressional Research Service and CMS. "Contested" determinations exclude defaults and include cases in which both parties submitted offers. Certified IDR entity fees are charged separately from the federal administrative fee and may vary by entity and batching structure. The author's contingency fee reflects one personal contractual arrangement and is not presented as representative of industry pricing. QPA: qualifying payment amount; IDR: independent dispute resolution; CRS: Congressional Research Service; CMS: Centers for Medicare and Medicaid Services

Metric	Value	Source
Provider/facility prevailing rate, all 2024 determinations	~85%	CRS R48738 [2]
Provider prevailing rate, contested (nondefault) determinations	~85%	CRS R48738 [2]
Selected offers exceeding the QPA	~85%	CRS R48738 [2]
Insurer offers at or below the QPA	~47%	CRS R48738 [2]
Median prevailing offer vs. QPA, emergency-service disputes (2023-2024)	~2.5-3×	CRS R48851 [3]
14-state sample: median QPA vs. median 2025 in-network rate	QPA slightly higher	CRS R48851 [3]
14-state sample: median prevailing offer vs. median in-network rate	>3×	CRS R48851 [3]
Certified IDR entity fee, single determination	$200 to $840	CMS fee fact sheet [4]
Certified IDR entity fee, batched determination	$268 to $1,173	CMS fee fact sheet [4]
Author’s IDR contingency fee (personal disclosure, not representative)	30% initial → 25% current	Author disclosure

Why the QPA warrants independent examination

An in-network rate is the product of a bargain. A provider may accept a discounted payment in exchange for network inclusion, patient volume, contractual predictability, and administrative arrangements. A nonparticipating provider has not accepted that bargain for the disputed service. Importing network pricing into a noncontracted transaction may be administratively convenient, but it is not neutral. It is network pricing without a network contract.

The QPA's formula is established by statute and regulation; insurers do not possess unlimited discretion to invent the number [1,2]. The structural concern is narrower and stronger. The insurer applies the prescribed methodology to contract data the provider cannot independently inspect, calculates a benchmark affecting the dispute, issues an initial payment or denial that need not equal that benchmark, and may then invoke the same benchmark to support its position in arbitration [2].

This creates an information asymmetry at the core of the process. The financially interested payer possesses the underlying contract universe; the noncontracted provider sees only the reported result [2]. Without a practical mechanism for independent verification, providers, arbitrators, policymakers, and the public cannot know whether a disputed QPA reflects the correct contracts, specialty, geography, modifiers, and aggregation rules. A benchmark that cannot be checked by the party forced to contest it should not receive automatic deference.

Recent federal analysis adds an important complication. In a 14-state sample of emergency-service disputes, the median QPA was slightly higher than the median 2025 in-network rate among IDR-participating providers, while the median prevailing offer exceeded three times that in-network rate [3]. This finding weakens any simplistic claim that every divergence proves QPA undercalculation. It also reinforces the need to examine the full chain, QPA construction, insurer offer strategy, provider offer strategy, statutory factors, and arbitrator selection, rather than treating any single number as self-validating.

The correct policy response is not to declare every QPA invalid or every award justified. It is to stop treating an unverifiable benchmark as presumptively sufficient while offers anchored to it are repeatedly not selected. The burden of explanation should not fall only on the physicians who prevail; it should also fall on the benchmark and on payer offers that are not selected after neutral review.

The cost of reaching the remedy

The IDR process is not a simple appeal portal. Parties must first complete a 30-business-day open-negotiation period, and initiation of IDR is generally subject to a short filing window after that period closes [2]. Eligibility depends on plan type, jurisdiction, service, geography, coding, and batching rules. Missing the procedural window can end the claim before anyone asks whether the payment was adequate. The care remains real. The physician's expense remains real. Only the remedy disappears.

The growth of third-party IDR representatives is therefore unsurprising. CRS documented increasing use of such representatives during 2024 and noted that they may allow smaller providers to participate by absorbing the administrative burden [2]. The author's own IDR services agreement initially required 30% of recoveries and currently requires 25%. That single contract is not an industry estimate. It is a concrete example of what a physician may have to surrender simply to use the remedy Congress created.

Formal fees add another barrier. Certified IDR entity fees range from $200 to $840 for a single determination and from $268 to $1,173 for a batched determination, with additional charges permitted for larger batches [4]. A complex operation may involve multiple disputed service lines. When separate determinations are required or strategically necessary, fees can accumulate into thousands of dollars before a neutral entity reviews the merits.

This matters to interpretation of the data. Claims reaching IDR are filtered by deadlines, expertise, expected recovery, and willingness to bear transaction costs. Selection bias is real. But the same friction undermines the caricature of IDR as an effortless mechanism for converting inflated charges into physician income. By the time a claim reaches determination, the physician may already have delivered and financed the care, waited through negotiation, paid an intermediary, and advanced arbitration fees merely to reach neutral review [4].

A binding award without reliable enforcement

A favorable determination should end the dispute. Federal rules require payment within 30 business days [1]. Yet the enforcement architecture remains incomplete. In Guardian Flight, L.L.C. v. Health Care Service Corporation, the United States Court of Appeals for the Fifth Circuit held that the NSA does not create a private right of action for providers seeking to enforce unpaid IDR awards [5]. A physician can therefore provide the care, navigate the process, win the case, and still lack a direct federal collection action.

Public data do not establish the national prevalence or duration of post-award nonpayment, and this editorial does not claim that delayed payment is universal. The narrower point is enough: a binding federal determination may leave the prevailing provider without a direct federal collection action in at least one appellate jurisdiction.

That gap should be measured and corrected. A system that publishes award totals but not carrier-level payment timing celebrates adjudication while obscuring compliance. Every payment retained beyond the statutory deadline shifts financing pressure from the payer to the medical practice and functions, in practical terms, as an interest-free loan.

Award totals are not physician profit

Public discussion often treats aggregate IDR awards as though they were net physician earnings. They are not. An award is gross reimbursement for care already delivered, not net physician income. Depending on the prevailing party, it may support professional-practice overhead or facility costs, including staffing, malpractice coverage, supplies, billing, compliance, financing, and substantial intermediary expenses. The physician did not create a new expense by prevailing in arbitration; the patient had already been treated, and the dispute concerned the amount payable under the governing federal process.

Insurer assertions that IDR contributes to premium growth should be tested, not repeated as self-proving [3]. The relevant quantity is incremental IDR expense above what the payer would otherwise have paid, measured against commercial premium revenue and medical claims expense. Aggregate award totals alone do not answer that question. Nor does insurer profitability. The required analysis is carrier-specific and should separate gross payment, incremental payment above the payer offer, administrative fees, and payment compliance.

Three reforms that preserve patients and improve evidence

First, QPA inputs should be independently verifiable. Regulators or an independent auditor should have confidential access to the contract-level data needed to confirm that QPAs were calculated using the correct contracts, specialty, geography, modifiers, and aggregation rules. Public disclosure of proprietary contract terms is unnecessary; credible verification is not.

Second, the Centers for Medicare and Medicaid Services (CMS) should replace massive quarterly data tranches as the sole practical form of transparency with a searchable, carrier-level database. At minimum, it should report QPAs, payer offers, provider offers, selected amounts, default status, determination fees, and payment timing. Data technically available only to analysts with specialized software are disclosed, but they are not meaningfully transparent. The public should not need a data science team to evaluate claims being used to redesign a national payment system.

Third, post-award compliance should carry automatic consequences. Awards unpaid after the statutory deadline should accrue interest, and repeated or unjustified nonpayment should trigger proportionate civil penalties after notice and an opportunity to correct genuine administrative errors. A binding decision without dependable enforcement is only partially binding in practice.

These reforms do not restore balance billing, guarantee provider prices, or shield high-volume filers and implausible awards from scrutiny. They preserve patient protections while making the replacement payment system auditable, accessible, and enforceable. That is not a physician windfall. It is the minimum architecture of a credible dispute-resolution system.

Conclusion

Patient protection and payment valuation are separate policy questions. Protecting patients from unexpected bills does not require automatic deference to any payment benchmark.

A credible dispute-resolution system should be independently verifiable, meaningfully transparent, accessible to both parties, and capable of enforcing its final determinations. These principles do not predetermine the appropriate payment in any individual dispute. They establish the minimum safeguards necessary for confidence in the process.

Protect the patient. Preserve neutral review. Verify the benchmark. Enforce the award.
